# Catastrophic health expenditure and its determinants in households with lung cancer patients in China: a retrospective cohort study

**DOI:** 10.1186/s12885-021-09030-w

**Published:** 2021-12-10

**Authors:** Cheng-yao Sun, Ju-fang Shi, Wen-qi Fu, Xin Zhang, Guo-xiang Liu, Wan-qing Chen, Jie He

**Affiliations:** 1 Department of Health Economics, College of Health Management of Harbin Medical University, 157 Baojian Road, Harbin, People’s Republic of China; 2 Office of Cancer Screening, National Cancer Center / National Clinical Research Center for Cancer / Cancer Hospital, Chinese Academy of Medical Sciences and Peking Union Medical College, Beijing, 100021 China; 3 National Cancer Center/National Clinical Research Center for Cancer/Cancer Hospital, Chinese Academy of Medical Sciences and Peking Union Medical College, Beijing, China

**Keywords:** Catastrophic health expenditures, Insurance, Lung cancer, China

## Abstract

**Background:**

Numerous studies have examined catastrophic health expenditures (CHE) worldwide, mostly focusing on general or common chronic populations, rather than particularly vulnerable groups. This study assessed the medical expenditure and compensation of lung cancer, and explored the extent and influencing factors of CHE among households with lung cancer patients in China.

**Methods:**

During 2018–2019, a hospital-based multicenter retrospective survey was conducted in seven provinces/municipalities across China as a part of the Cancer Screening Program of Urban China. CHE was measured according to the proportion of out-of-pocket (OOP) health payments of households on non-food expenditures. Chi-square tests and logistic regression analysis was adjusted to determine the factors that significantly influenced the likelihood of a household with lung cancer patient to incur in CHE.

**Results:**

In total, 470 households with lung cancer patients were included in the analysis. Health insurance was shown to protect some households from the impact of CHE. Nonetheless, CHE incidence (78.1%) and intensity (14.02% for average distance and 22.56% for relative distance) were still relatively high among households with lung cancer patients. The incidence was lower in households covered by the Urban Employee Basic Medical Insurance (UEMBI) insurance, with higher income level and shorter disease course.

**Conclusion:**

More attention is needed for CHE incidence among vulnerable populations in China. Households with lung cancer patients were shown to be more likely to develop CHE. Therefore, policy makers should focus on improving the financial protection and reducing the economic burden of this disease.

## Introduction

Some of the fundamental roles of a healthcare system are protecting families from disease-related financial catastrophe and achieving health equality [1]. Hence, a high incidence of catastrophic health expenditures (CHE) means that a given health system is not achieving its goal of financial protection provision. In this context, a retrospective observational study conducted in 133 countries found that, within 5 years (i.e., from 2005 to 2010), CHE rose from 9.7 to 11.7% [2]. Another survey covering 89 countries worldwide showed that 150 million people face CHE annually, with low and middle income countries (LMICs) showing higher levels of CHE than high income countries [3]. Moreover, the World Health Survey showed that the incidence of CHE in developed countries, such as the United States of America and Germany, was < 1%, while that in developing countries, such as Vietnam and Brazil, was > 10% [4]. Like many developing countries, China also faces a high disease burden and out-of-pocket (OOP) healthcare expenditure is relatively high; the incidence of CHE was about 8.94% in 2016 [5].

Chronic diseases often come with high economic burdens. Many studies have shown that families with members who have chronic disease face higher financial risks than other families. In Bangalore, India, a study showed that CHE incidence in families with members who have chronic disease was 16%, significantly exceeding the average level of the general population [6]. In rural China, families with members who have hypertension had 1.7–2.6 times higher occurrence of CHE than families without members with non-communicable diseases (NCDs) [7]. Another study reported that in China the incidence of CHE in families with members who have hypertension with other NCDs was 46.9% in 2013 [8], which significantly exceeded the average of 8.94%.

Studies on CHE and its determinants have been conducted globally. However, most have focused on the whole population or common chronic populations, rather than on particularly vulnerable groups, such as cancer patients. In 2018, GLOBOCAN statistics found that, worldwide, there were 18.1 million new cancer cases and 9.6 million cancer deaths, with lung cancer being the most commonly diagnosed cancer and the leading cause of cancer death [9]. There were 3.804 million new cancer cases and 2.296 million deaths in 2014 across China, with lung cancer similarly being the most commonly diagnosed cancer (20%) and the leading cause of cancer death (27.3%) [10]. Owing to the high burden of morbidity and mortality related to lung cancer in China, we infer that it can give rise to CHE and impose a substantial financial burden in the Chinese population.

Health insurance has been widely accepted as an effective strategy to prevent CHE [11]. By 2015, more than 95% of Chinese citizens participated in social health insurance [12]. The three types of social health insurance subsidized by the Chinese government are: Urban Employee Basic Medical Insurance (UEBMI), Urban Resident Basic Medical Insurance (URBMI), and the New Cooperative Medical Scheme (NCMS). The UEBMI was launched in 1998. Employers and employees jointly pay insurance premiums, and it covers urban employees and retirees in the formal sector, including those who previously enjoyed free medical care in public institutions and state-owned enterprises. The NCMS and URBMI were launched in 2003 and 2007, respectively. The former covers rural residents, and the latter covers urban residents who are not eligible for UEBMI, such as unemployed individuals and children. The reimbursement rates are the highest for the UEBMI, followed by the URBMI, and the NCMS [13].

Accordingly, the objective of this study was to assess the medical expenditure and compensation of lung cancer and explore the extent and influencing factors of CHE among households with lung cancer patients in China. Furthermore, this study evaluated the financial protection capacity of health insurance for families with lung cancer patients. Our findings may contribute to improving and adjusting related medical insurance policy, helping to further relieve the economic burden of this critical disease.

## Methods and materials

### Data source

The Cancer Screening Program in Urban China (CanSPUC), which was supported by the National Health and Family Planning Commission (NHFPC) in China, has been a crucial cancer prevention initiative that began in August 2012 [14]. The primary objective of the CanSPUC was to explore an appropriate implementation approach for screening of population at high risk and early diagnosis of major cancers in urban populations in China, promoting the use of mature screening and early diagnosis technology for common cancers to reduce mortalities. The program covered 13 provinces/municipalities in China [including eastern (Beijing, Hebei, Liaoning, Jiangsu, Zhejiang, Shandong, and Guangdong), central (Heilongjiang, Henan, and Hunan), and western regions (Chongqing, Gansu, and Xinjiang) [15] by 2014.

A multicenter cross-sectional survey was conducted from January 2018 to June 2019 as part of CanSPUC. Geographic regions/provinces were grouped into eastern, central and western in line with the classification of economic development zones by the Chinese National Bureau of Statistics. Nine tertiary hospitals, were selected from these zones considering cancer patient volumes and completeness of medical records, including Guangdong Cancer Hospital (eastern); Anhui Cancer Hospital, Heilongjiang Cancer Hospital, Shanxi Cancer Hospital (central); Guangxi Cancer Hospital, Yunnan Cancer Hospital, the Regional Cancer Hospital and two city hospitals in Inner Mongolia (western).

Cancer patients initially diagnosed between 01 January 2015 and 31 December 2016 were eligible for this study (including lung, female breast, colorectal, esophageal, gastric, and liver cancers). Eligible study participants were identified from the hospital records and then approached for a survey. Upon consent to participate in the study, an informed consent form was completed by the patient. The questionnaire was administered through face-to-face interviews. The survey was coordinated by the National Cancer Center. The interviewers were trained prior to deployment and required to check completeness of the questionnaire before concluding each interview. The questionnaire for this survey collected data regarding demographic characteristics, household income and expenditure, medical expenses for cancer treatment, and insurance compensation. A total of 2565 patients being investigated.

Regarding household income and expenditure, respondents were asked to describe them for both 2015 and 2016. Regarding medical expenses for cancer treatment, when the course of the patient’s disease was 1 year, the respondents were asked to describe this variable over a one-year period (i.e., 2 months before and 10 months after the diagnosis); when the course of the patient’s disease was 2 years, the respondents were asked to describe this variable over a two-year period (i.e., 2 months before and 22 months after the diagnosis); these costs included payments for hospital diagnosis, treatment, and medicines (prescription and non-prescription drugs) purchased from pharmacy retail stores.

Among these, lung cancer patients were selected as subjects of this study. Inclusion criteria were as follows: (1) having been diagnosed for the first time with primary lung cancer, (2) having been initially diagnosed between 1 January 2015 and 31 December 2016, and (3) having subsequently received cancer treatment. The exclusion criterion was having cancers in multiple organs. Regarding household income and expenditure, owing to difficulties in articulating a clear cut-off point for the income and expenditure data, we calculated average income and expenditure across the 2 years to match the lung cancer treatment cost data. Regarding medical expenses, when the course of disease was 2 years, we calculated the average medical expenses across 2 years. Chinese Renminbi (RMB) was converted into US dollars based on the average 2015 exchange rate (Chinese RMB 6.2284 yuan = $US1.00).

Data were double-entered into EpiData 3.1 to ensure accuracy and analyzed using Stata 15.

### Measuring CHE incidence

Two thresholds have been widely used to define CHE: OOP healthcare expenditure greater than or equal to 10% of total household expenditure [16, 17]; or a non-food household expenditure greater than or equal to 40% [18, 19]. We measured CHE using the indicators reported by Adam et al. [20]. In this study, OOP health expenditure only covered direct medical expenses, excluding direct non-medical (e.g., transportation and nutrition) and indirect costs. To calculate CHE, we used non-food household expenditure as the denominator, thereby partly avoiding measurement bias found in other methods that often cause the neglect of low income families. In the equation below, the indicator *Ei* defined whether CHE occurred:

1 $${E}_i=\left\{\begin{array}{c}0,\kern0.5em \frac{oop}{e_i-{f}_i}<z\ \\ {}1,\kern0.5em \frac{oop}{e_i-{f}_i}>z\end{array}\right.$$

where e_i_ is the total consumption expenditure of household i, and f_i_ is the food expenditure of household i. Before insurance compensation, the OOP is the total medical cost (including insurance compensation). The e_i_ is the total consumption expenditure of household i (including insurance compensation). After insurance compensation, the OOP is the patient’s medical costs after reimbursement. The e_i_ is the total household expenditure of household i after insurance compensation is deleted. z is the threshold of CHE, which we set to 0.4. CHE incidence and intensity were estimated as follows:

2 $$\mathrm{H}=1/\mathrm{N}{\sum}_{i=1}^N Ei$$

3 $$\mathrm{O}=1/\mathrm{N}{\sum}_{i=1}^N\left(\left(\frac{oop}{ei- fi}\right)-z\right)\ast Ei$$

4 $$\mathrm{MPO}=1/\mathrm{Na}{\sum}_{i-1}^{Na}\left(\left(\frac{oop}{ei- fi}\right)-z\right)\ast Ei$$

where N is the sample size; Na is the sample size of households incurring in CHE; H is CHE incidence; O is the average distance; and MPO is the mean relative distance. CHE intensity was calculated using average distance and mean relative distance: average distance measures the degree by which an average OOP health expenditure exceeds the given CHE threshold of all lung cancer families; and mean relative distance represents CHE intensity in families suffering from CHE.

### Statistical analysis

Medical expenditure, insurance compensation, household income and expenditure, and OOP were presented using means and standard deviations. The analysis of the collected data was done while stratifying the variables by two different insurance groups: the UEBMI group and the Urban and Rural Resident Basic Medical Insurance (U&RRBMI) group (U&RRBMI includes URBMI and NCMS).

We used the chi-squared test to compare the CHE incidence in lung cancer families before and after insurance compensation. In addition, the chi-squared test was applied to examine the associations between CHE and other variables including gender, age, education level, household size, insurance, and income level. Additionally, multivariate logistic regression analyses were performed and *P* < .05 was considered statistically significant.

Econometricians usually dismissed sample size problems based on the strength of the asymptotic quality of the standard maximum likelihood (ML) model [21]. However, others made some suggestions regarding sample size; Hart and Clark found that reasoning problems began to occur when the sample size was less than 30. In another study with a sample of 200 participants, Hart concluded that this sample produced a consistent estimate of the probit model [22]. Eliaison additionally recommended that the sample size should be more than 60 [23]. Therefore, to meet the model requirements, we deemed a sample size of 470 lung cancer families to be adequate.

## Results

### Sample characteristics

A total of 470 households with a lung cancer patient were recruited. Table 1 shows the general characteristics of lung cancer patients and their families. In total, 63% of the lung cancer patients were male and the sample showed a rather high average age at 63.6. Regarding education, most graduated from junior high school and below (61.3%), with 38.7% having graduated from senior high school. Moreover, 86.6% of the respondents were married. The course of disease revealed that 83.2% had cancer for 1–2 years. Regarding household size, 59.4% of the lung cancer families had less than 4 people and 35.7% had 4–6 people, together totalizing 95.1%. Regarding type of health insurance, most respondents were covered by health insurance policies, with UEBMI accounting for 54.7% and U&RRBMI for 44%.

**Table 1:** Participant characteristics

Sociodemographic Characteristics	Number	Percent (%)
**Gender**		
Male	296	63.00
Female	174	37.00
**Age (years)**		
≤ 60	186	39.60
> 60	284	60.40
**Education level**		
Junior high school and below	288	61.30
Senior high school and above	182	38.70
**Marital status**		
Married	407	86.60
Rest^1^	63	13.40
**Course of disease**		
< 1 year	79	16.80
1–2 year	391	83.20
**Household size**		
≤ 3	279	59.40
4–6	168	35.70
≥ 7	23	4.90
**Insurance**		
UEBMI	257	54.70
U&RRBMI	207	44.00
Rest^2^	6	1.30
**Income level(80000RMB)**		
≤ 12844USD	346	73.60
>12844USD	124	26.40

Rest 1 represents unmarried, divorced and widowed. Rest 2 stands for public medical, commercial insurance and uninsured. *Abbreviations*: The *UEBMI* represents Urban Employee Basic Medical Insurance. The U&RRBMI represents Urban and Rural Resident Basic Medical Insurance

The mean annual household expenditure of the UEBMI and U&RRBMI groups were $11,366 (SD = 8255) and $9835 (SD = 8993), respectively (Table 2). The annual medical household expenditure of the UEBMI and U&RRBMI groups were $13,527 (SD = 10,265) and $12,689 (SD = 8962), respectively. The average insurance compensation provided to the UEBMI and U&RRBMI groups were $6633 (SD = 6038) and $4671 (SD = 3888), respectively. The mean OOP expenditure for lung cancer care of the UEBMI and U&RRBMI groups were $6674 (SD = 6519) and $7892 (SD = 6967), respectively.

**Table 2:** Medical expenditure, compensation and OOP payment by insurance type

	UEBMI		U&RRBMI		Total	
Indicators	Mean	SD	Mean	SD	Mean	SD
Medical expenditure	13,527	10,265	12,689	8962	13,173	9677
Insurance reimbursement	6633	6038	4671	3888	5755	5258
OOP payment	6674	6519	7892	6967	7242	6768
Household income	14,362	11,607	7907	8248	11,473	10,697
Household expenditure	11,366	8255	9835	8993	10,733	8618
Food expenditure	3027	2277	1872	1855	2509	2167

*Abbreviations*: The *UEBMI* represents Urban Employee Basic Medical Insurance. The U&RRBMI represents Urban and Rural Resident Basic Medical Insurance

### CHE and the financial protection capacity of health insurance

**Fig. 1:**
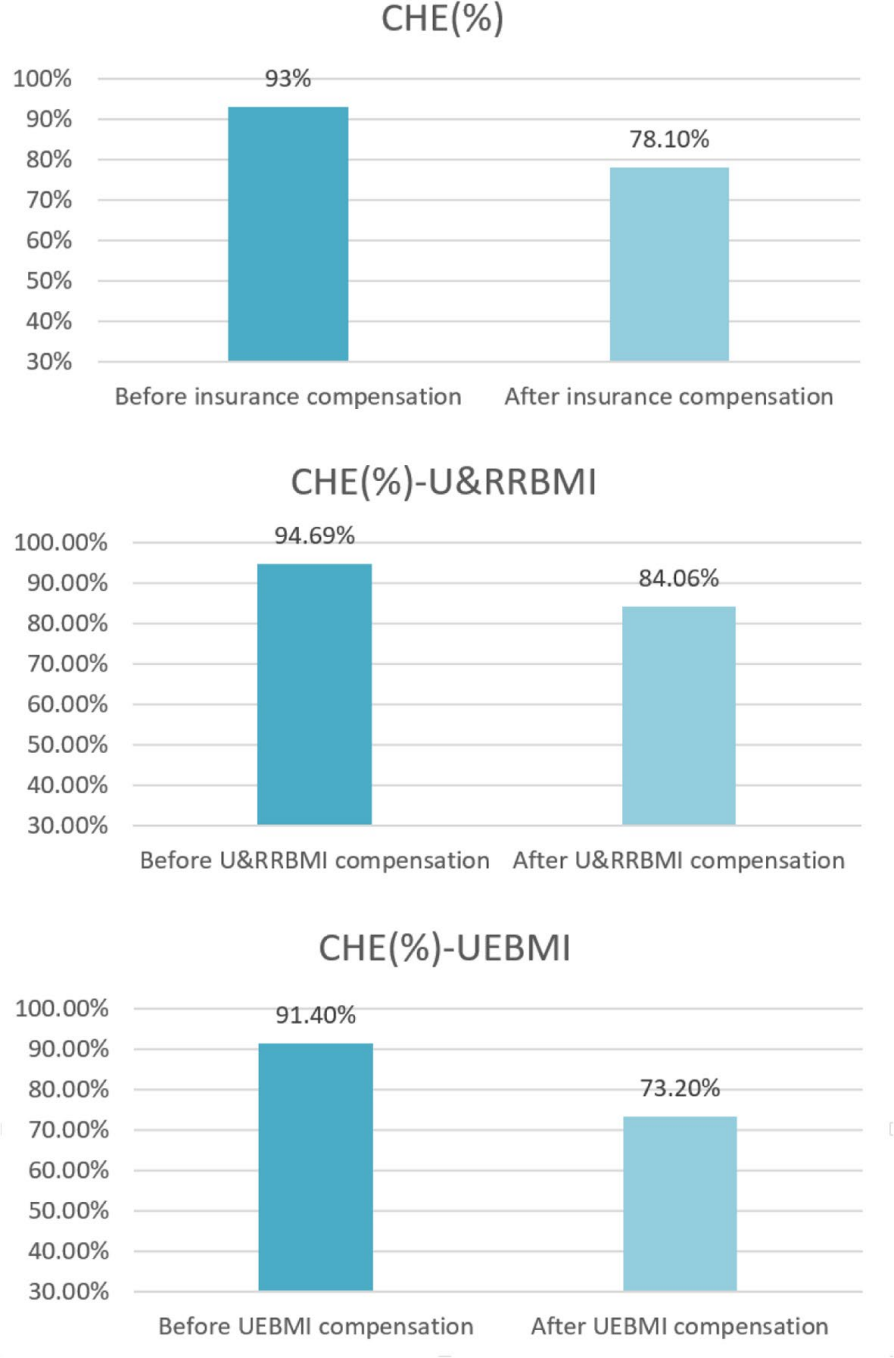
The CHE incidence before and after insurance compensation

Figure 1 and Table 3 show the CHE incidence and intensity of lung cancer families before and after insurance compensation. The CHE incidence of lung cancer families before insurance compensation was 93 and 78.10% (*p* = 0.000) after insurance compensation, indicating that insurance compensation reduced CHE incidence by 14.9%. Meanwhile, the CHE incidence of lung cancer families covered by UEBMI were 91.4 and 73.2% (*p* = 0.000) before and after insurance compensation, respectively; namely, UEBMI insurance compensation reduced CHE incidence by 18.2%. The CHE incidence of lung cancer families covered by U&RRBMI were 94.69 and 84.06% (*p* = 0.000) before and after insurance compensation, respectively; hence, U&RRBMI insurance compensation reduced the incidence of CHE by 10.63%.

**Table 3:** the CHE incidence and intensity before and after insurance compensation

	Average distance	Reduced	Relative distance	Reduced
**Total**				
Before compensation	23.80%		27.70%	
After compensation	14.02%	9.78%	22.56%	5.14%
**UEBMI**				
Before compensation	21.68%		26.53%	
After compensation	10.96%	10.72%	10.86%	15.67%
**U&RRBMI**				
Before compensation	26.28%		28.94%	
After compensation	17.68%	8.6%	24.08%	4.86%

*Abbreviations*: The *UEBMI* represents Urban Employee Basic Medical Insurance. The U&RRBMI represents Urban and Rural Resident Basic Medical Insurance

The relative distance was 22.56% after insurance compensation, indicating that families which suffered from CHE had an average of 62.56% of their household expenditure net of food spending characterized by OOP expenditure. Furthermore, the relative distance was 10.86% in lung cancer families covered by UEBMI (i.e., this insurance reduced the relative distance by 15.67%) and 24.08% in those covered by U&RRBMI (i.e., this insurance reduced the relative distance by 4.86%). The average distance of lung cancer families was 14.02% after insurance compensation, with 10.96% for lung cancer families covered by UEBMI and 17.68% for those covered by U&RRBMI.

Patients with lung cancer who had longer course of disease, U&RRBMI insurance, lower income level tended to have higher incidence of CHE than the others (Table 4). The logistic regression model further confirmed that course of disease, insurance, age, and income level were significant predictors of CHE. (Table 5).

**Table 4:** Incidence of CHE after insurance compensation

Sociodemographic Characteristics	CHE(n)	Percent (%)	*P*
Gender			
Male	236	79.73%	0.261
Female	131	75.29%	
Age (years)			
≤60	142	76.34%	0.460
>60	225	79.23%	
Education level			
Junior high school and below	229	79.51%	0.346
Senior high school and above	138	75.82%	
Marital status			
Married	319	78.38%	0.696
Rest1	48	76.19%	
Course of disease			
<1 year	44	55.70%	0.000
1–2 year	323	82.61%	
Household size			
≤3	218	78.14%	0.580
4--6	133	79.17%	
≥7	16	69.57%	
Insurance			
UEBMI	188	73.15%	0.018
U&RRBMI	174	84.06%	
Rest2	5	83.33%	
Income level(80,000 RMB)			
≤12844USD	289	83.53%	0.000
>12844USD	78	62.90%	

P based on chi-square test. *Abbreviations*: The *UEBMI* represents Urban Employee Basic Medical Insurance. The U&RRBMI represents Urban and Rural Resident Basic Medical Insurance

**Table 5:** Multivariate logistic regression model of determinants of CHE

Determinants	OR	SE	p	95% CI	
**Gender (ref male)**					
Female	0.874	0.225	0.599	0.528	1.446
**Age (years,ref ≤ 60)**					
>60	2.162	0.591	0.005	1.266	3.693
**Education level (ref Junior high school and below)**					
Senior high school and above	1.278	0.365	0.390	0.730	2.239
Marital status (ref Married)					
Rest^1^	0.827	0.302	0.604	0.404	1.693
**Course of disease (ref <1 year)**					
1–2 year	5.692	1.725	0.000	3.143	10.308
**Household size (ref ≤ 3)**					
4–6	1.024	0.279	0.930	0.601	1.746
≥7	0.444	0.238	0.129	0.155	1.268
**Insurance (ref UEMBI)**					
U&RRBMI	2.262	0.664	0.005	1.272	4.023
Rest2	1.656	1.948	0.668	0.165	16.612
**Income level (ref ≤ 12,844 USD)**					
>12,844 USD	0.292	0.081	0.000	0.170	0.503
**Constant**	0.637	0.261	0.272	0.285	1.423

Rest 1 represents unmarried, divorced and widowed. Rest 2 stands for public medical, commercial insurance and uninsured. *Abbreviations*: The *UEBMI* represents Urban Employee Basic Medical Insurance. The U&RRBMI represents Urban and Rural Resident Basic Medical Insurance

## Discussion

To the best of our knowledge, this was the first study to analyze the impact of insurance on and the extent and determinant factors of CHE, as well as the medical expenditure and insurance compensation in households with members who have lung cancer patients in China.

Our findings showed that the mean medical expenditure of lung cancer patients was $13,173, which is much higher than the gross domestic product (GDP) per capita in China ($7904 in 2015) [24]. The average OOP payment after insurance compensation for lung cancer patients was $7242, being nearer to the aforementioned GDP per capita. The medical expenditure of those covered by UEBMI was higher than that of those covered by U&RRBMI, albeit the OOP payment of the first was lower than that of the second; this owes to the higher percentage of insurance reimbursement of the UEBMI insurance. The household expenditure was higher in those covered by UEBMI than by U&RRBMI. Therefore, OOP health expenditure brought about a huge financial burden to the households with members who have lung cancer, especially those covered by U&RRBMI insurance. This finding is consistent with previous studies [25, 26].

Additionally, we found that 78.10% of the households with lung cancer patients demonstrated health expenditure that went above 40% of their non-food expenditures. In another Chinese study, the overall CHE incidence was 13.0% (the threshold is set to 40%) [27]. In another study, 21.5% of the families with members who had only hypertension and 46.9% of the families with members who had hypertension plus other NCDs incurred in CHE [8]. Namely, in China, lung cancer households’ risk tolerance for health expenditure may be much lower than the average for the general population or for households with other chronic diseases.

Moreover, our findings showed that the studied households had a lower CHE incidence after insurance compensation than before it; namely, the studied health insurance systems (i.e., UEMBI and U&RRBMI) protected some households from the impact of CHE. We also found that the impact of UEMBI on CHE was greater than that of U&RRBMI. However, even if provided relevant data on the difference in compensation levels between these two insurances, we still see a need for further study on the overall incidence of CHE in lung cancer families; given that our findings showed that lung cancer families may be one of the most vulnerable groups to CHE, future research should pay more attention to such households and study the incidence of this issue.

In this study, increased disease course, low-level insurance, age over 60 years, and low-level income significantly impacted CHE; these findings find consonance in the literature. A study examining the determinants factors of CHE among cancer families across 10 countries in Southeast Asia found that income level, education, and type of health institution and health insurance are influencing factors of CHE [28], hence corroborating our findings. Another study demonstrated that Chinese older adults’ risk tolerance for healthcare payments was actually lower than the average in China [29]. Therefore, to help mitigate CHE risk in households with lung cancer patients, we suggest for the central government to adjust the related policies by age, as there seems to be age differences on the topic.

In 2009, China’s health reforms focused on reducing OOP spending. Catastrophic medical insurance was introduced in 2012 to cover more than 4 million patients with catastrophic illnesses [30]. However, CHE for lung cancer patients remains high even after the implementation of catastrophic insurance. According to our findings, the CHE rates for households with lung cancer patients leads to high medical costs. The rapid growth of the national health expenditure per capita is an important cause of CHE. The National Bureau of Statistics showed that the national health expenditure per capita increased from 1314 Yuan in 2009 to 4236 Yuan in 2018 [31]. Therefore, policy-makers should endeavor to develop policies that help control the cost of medical procedures. Additionally, China’s catastrophic medical insurance is operated by commercial insurance companies. The catastrophic medical insurance is not perfect and has not played the role it should have played.

Chinese hospitals are organized according to a three-tier system that recognizes the ability to provide medical care, education, and conduct medical research [15]. Based on this system, hospitals are designated as primary, secondary, or tertiary institutions, and the medical expenses tend to be higher in high-level hospitals. However, owing to the limited capacity of medical care in primary hospitals, many patients choose to go to high-level hospitals. Therefore, to reduce patients’ financial burden related to health expenditure, the resource allocation of the three-tier system should be optimized and aim to avoid congregating patients in tertiary hospitals.

Some limitations of our study must be acknowledged. First, we evaluated CHE incidence and intensity only in households with lung cancer patients that actually presented themselves for treatment, not considering households that did not receive treatment. Many patients decide not to receive treatment owing to, for instance, insufficient family funds or thinking that the treatment will not save their lives. Second, OOP health expenditure in this study only covered direct medical expenditures, excluding direct non-medical (e.g., transportation and nutrition) and indirect costs. Therefore, CHE incidence might have been underestimated to some extent. Third, the sample size of lung cancer households was not very large. Therefore, it is recommended for future studies to recruit a larger sample. Fourth, the severity of lung cancer is also the impact factor of financial burden [32]. In the future, we will take into account variables related to disease severity such as cancer staging and treatment methods to explore the influencing factors of CHE in a more comprehensive manner. Finally, the findings of this study were based on patients in tertiary hospitals in an urban setting; hence, the conclusion should not be overstated.

## Conclusions

The findings revealed that CHE incidence and intensity were relatively high among households with lung cancer patients. Furthermore, more attention is warranted to households covered by U&RRBMI because they seemed to be, based on our findings, at the highest risk of incurring CHE. Moreover, some social factors significantly affected CHE, meaning that policies aimed at reducing CHE must consider some of the described social factors of households and patients.

## Data Availability

The data that support the findings of this study are available from Chinese Academy of Medical Sciences. The datasets used in this study were available from the corresponding author upon reasonable request.

## Abbreviations

CHE: Catastrophic health expenditures
OOP: Out-of-pocket
UEMBI: Urban Employee Basic Medical Insurance
NCDs: Non-communicable diseases
URBMI: Urban Resident Basic Medical Insurance
NCMS: New Cooperative Medical Scheme
CanSPUC: The Cancer Screening Program in Urban China
NHFPC: National Health and Family Planning Commission
RMB: Renminbi
MPO: Mean relative distance
O: Average distance
U&RRBMI: Urban and Rural Resident Basic Medical Insurance
GDP: Gross domestic product
